# Association between Aqueous Humor Cytokines and Structural Characteristics Based on Optical Coherence Tomography in Patients with Diabetic Macular Edema

**DOI:** 10.1155/2023/3987281

**Published:** 2023-02-07

**Authors:** Yan Chen, Bin Zheng, Haidong Li, Li Lin, Yuanyuan Fan, Mengai Wu

**Affiliations:** National Clinical Research Center for Ocular Diseases, Eye Hospital, Wenzhou Medical University, Wenzhou, China

## Abstract

**Purpose:**

To investigate the relationship between aqueous humor cytokines and structural characteristics based on optical coherence tomography (OCT) in patients with diabetic macular edema (DME).

**Methods:**

Forty eyes of 28 patients with DME diagnosed in the Affiliated Eye Hospital of Wenzhou Medical University at Hangzhou were included. All patients collected aqueous humor during anti-VEGF treatment, and the IL-6, IL-8, IL-10, VEGF, VCAM-1, ICAM-1, TGF-*β*1, FGF, and MCP-1 concentrations were detected. OCT examination was performed before anti-VEGF treatment and 1 month after anti-VEGF operation. Central macular thickness (CMT), macular volume (MV), choroidal thickness (CT), and the number of hyperreflective foci (HRF) were obtained for analysis. Each eye was determined whether there is subretinal effusion (SRD), cystoid macular edema (CME), and diffuse retinal thickening (DRT).

**Results:**

The levels of IL-6 and FGF in DME patients with SRD were significantly higher than those without SRD (all *P* < 0.05). The level of VEGF in DME patients with CME was significantly higher than that in DME patients without CME (*P* = 0.005); IL-6, TGF-*β*1, and MCP-1 were significantly higher in DME patients with DRT than that without DRT (all *P* < 0.05). There was no significant correlation between aqueous humor cytokines and retinal thickness and retinal volume. However, the thinner the CT, the higher the level of aqueous humor cytokines IL-6 (*r* = −0.313, *P* = 0.049) and FGF (*r* = −0.361, *P* = 0.022). A multivariate linear regression analysis showed that IL-6 was significantly correlated with CT (*P* = 0.002) and SRD (*P* = 0.017), FGF was also significantly correlated with CT (*P* = 0.002) and SRD (*P* = 0.005), and TGF-*β*1 was correlated with triglycerides (*P* = 0.030) and HRF (*P* = 0.021).

**Conclusion:**

DME patients with significant macular cystoid edema changes may be related to high VEGF concentrations and thin CT; meanwhile, the presence of SRD or a high number of HRF on OCT macular scans in DME patients may indicate high levels of intraocular inflammatory factors. Thus, OCT morphology characteristics to some extent reflect intraocular inflammatory factors and VEGF levels and may guide treatment alternatives.

## 1. Introduction

Diabetic macular edema (DME) is one of the major causes of vision loss in diabetic patients. Vascular endothelial growth factor (VEGF) plays an important role in the development of DME; anti-VEGF drugs have become the first-line treatment for DME. However, 30%–40% of patients are still insensitive to anti-VEGF therapy [1, 2]. Both clinical and experimental studies have confirmed that in addition to VEGF, inflammatory factors such as interleukin-6 (IL-6), interleukin-8 (IL-8), interleukin-10 (IL-10), VEGF, vascular cell adhesion molecule-1 (VCAM-1), intercellular adhesion molecule-1 (ICAM-1), transforming growth factor-*β*1 (TGF-*β*1), fibroblast growth factor (FGF), and monocyte chemoattractant protein-1 (MCP-1) also play important roles in the pathogenesis of DME [3, 4]. Therefore, patients with DME who are insensitive to anti-VEGF therapy may be treated with dexamethasone implant alone or in combination. Hence, it has been suggested that intraocular cytokine levels could be used to predict the outcomes of anti-VEGF therapy and to enhance treatment accuracy [5, 6].

However, intraocular cytokine testing is an invasive test that may raise the risk of infection and is currently mainly gathered at the time of surgical treatment; thus, it offers limited guidance on treatment options. Optical coherence tomography (OCT), a noninvasive test that provides high-resolution images of retinal microstructures in a noncontact and rapid manner, plays an important role in the diagnosis and follow-up of DME. Depending on the OCT morphology and pathogenesis, DME can manifest in one of the three forms: subfoveal neuroretinal detachment (SRD), cystoid macular edema (CME), and diffuse retinal thickening (DRT) [7, 8]. This study aims to analyze the relationship between aqueous humor cytokines and OCT structures in patients with DME, and to estimate the intraocular cytokine levels in patients with DME before treatment, to give clinicians more information to make better treatment plans.

## 2. Methods

This study adhered with the Declaration of Helsinki and was approved by the Ethics Committee of the Optometry Hospital of Wenzhou Medical University (No: 2021-003-K-03-01). All patients were informed and signed written informed consents. Consecutive patients with outpatient diagnosed DME and received anti-VEGF treatments were collected from March 2021 to December 2021 at the Affiliated Eye Hospital of Wenzhou Medical University. Inclusion criteria were as follows: (1) age 18–80 years; (2) OCT recording of central macular thickness (CMT) > 250 *μ*m. Exclusion criteria were as follows: (1) previous history of ocular trauma and uveitis; (2) combined macular degeneration, glaucoma, iris neovascularization, and other eye diseases; (3) previous history of vitreous surgery; (4) patients who had undergone cataract surgery, anti-VEGF injection, laser therapy, and other treatments.

**Figure 1:**
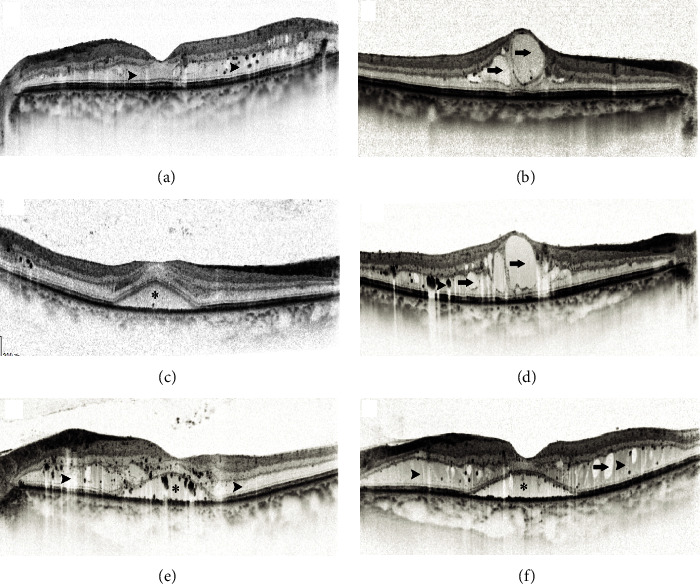
OCT images of different DME types. (a) DME primarily exhibits DRT, with macular spongy thickening indicated by arrowheads; (b) DME primarily exhibits CME, with intraretinal hyporeflective cystic cavity indicated by arrows; (c) DME primarily exhibits SRD, with an optically clear gap between retinal neuroepithelium and pigment epithelium indicated by asterisks; (d) DME exhibits CME and DRT; (e) DME exhibits DRT and SRD; (f) DME consists of a mixture of CME, DRT, and SRD types.

All patients underwent comprehensive ophthalmic examinations before anti-VEGF treatment, including best corrected visual acuity (BCVA), intraocular pressure, slit lamp microscopy, and dilated fundus examination. OCT three-dimensional macular scans were performed using Heidelberg OCT (Heidelberg Spectralis, Heidelberg, Germany) with 30° × 25° horizontal line scan mode, and the build in software was used to automatically calculate the CMT and macular volume (MV) at the end of the scan. Specifically, the macular area was divided into 1 circle and 2 annular areas according to the Early Treatment of Diabetic Retinopathy Study (ETDRS) Group division method, i.e., a 3 circular area with a diameter of 1 mm, 2 mm, and 3 mm centered on the fovea, and the mean CMT within a diameter of 1 mm circle and the mean MV within a diameter of 3 mm circle were calculated. OCT horizontal line scan centered at the macular with 100 frames per scan was also acquired to count hyperreflective foci (HRF) and measure choroidal thickness (CT). The intraretinal HRF was defined as isolated, well-defined foci with reflectance intensity not weaker than the retinal pigment epithelium (RPE) layer reflectance intensity, and with a diameter >20 *μ*m and <50 *μ*m. Two scorers manually counted HRF in the retina, disagreements between scorers were solved by open adjudication, and the mean value was taken for statistical analysis [9]. CT was defined as the distance between the outer border of the RPE and the inner border of the choroidal-scleral interface under the central macular. The presence of SRD, DRT, and CME was determined for each eye based on previous studies. SRD demonstrated as a plasmacytic separation between retinal neuroepithelium and pigment epithelium; CME was detected as intraretinal cystic hyporeflective lesion; DRT was identified as retinal spongy thickening and reduced intraretinal reflectivity (Figure 1).

The aqueous humor specimens were collected during anti-VEGF treatment, and 0.05–0.1 ml aqueous humor was extracted using a 30-gauge insulin syringe via limbal paracentesis before intravitreal injection. The sample was immediately transferred to a plastic tube and kept at −84 until assayed. Aqueous humor concentrations of IL-6, IL-8, IL-10, VEGF, VCAM-1, ICAM-1, TGF-*β*1, FGF, and MCP-1 were tested with CBA flex set (BD Cytometric Flex Set, San Diego, USA) with FACSCanto plus flow cytometer (BD Biosciences).

SPSS 26.0 statistical software was used to analyze the data. The Shapiro–Wilk test was used to evaluate the normality of data distribution. Non-normally distributed data were expressed as median ± quartiles, and the Mann–Whitney *U* test was used for comparison between groups. Normally, distributed data were expressed as the mean ± standard deviation, and the independent samples *t* test was used for comparison between the groups. The relationship between aqueous humor cytokines and retinal choroidal structures was analyzed by Spearman rank correlation. Aqueous humor cytokine data were log-transformed to reduce the effect of outliers; univariate and multiple linear regression analyses were performed to determine the correlation between variables. Variables with a significance of *P* < 0.15 in the univariate analysis were entered into the multivariate analysis. *P* < 0.05 was considered statistically significant.

## 3. Results

Forty eyes of 28 patients with a mean age of 62.5 ± 9.3 years were included in this study. Among them, nine (22.5%) eyes exhibited SRD, thirty two (80%) eyes exhibited CME, and thirty (75%) eyes exhibited DRT. All DME eyes were grouped according to whether they presented with DRT, CME, and SRD, the baseline characteristics were compared between the groups in Table 1, the aqueous humor cytokine levels and OCT parameters were compared and shown in Table 2. There were no significant differences in baseline characteristics in eyes with and without SRD, however, IL-6 and FGF levels were significantly higher in eyes with SRD than those without SRD (all *P* < 0.05). Similarly, there were no significant differences in baseline characteristics in eyes with and without CME, however, VEGF levels were significantly higher in eyes with CME than those without CME (*P*=0.005). Meanwhile, the CT in DME patients with CME was significantly smaller than those without CME (*P*=0.042). There were no significant differences in baseline characteristics in eyes with and without DRT; however, TGF-*β*1 levels were significantly higher in eyes with DRT than those without DRT (*P*=0.039). There were no significant differences in CMT, MV among different types of DME; however, HRF was significantly higher in DME patients with SRD than those without SRD (*P* < 0.001), and HRF was higher in DME patients with DRT than those without DRT (*P* > 0.05).

**Figure 2:**
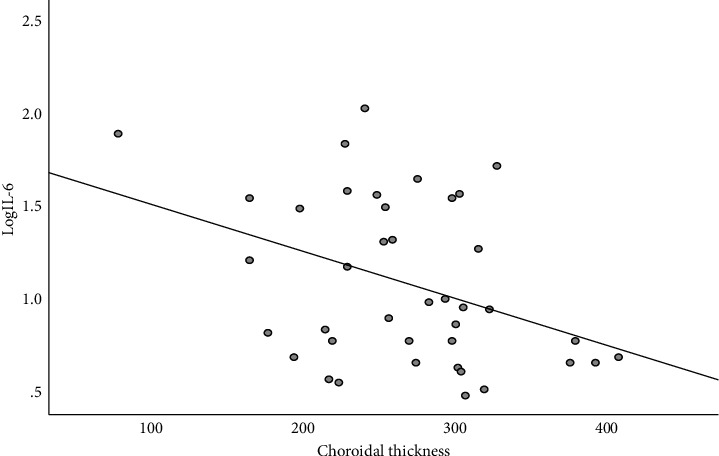
Relationship between aqueous humor IL-6 and CT in DME patients.

**Figure 3:**
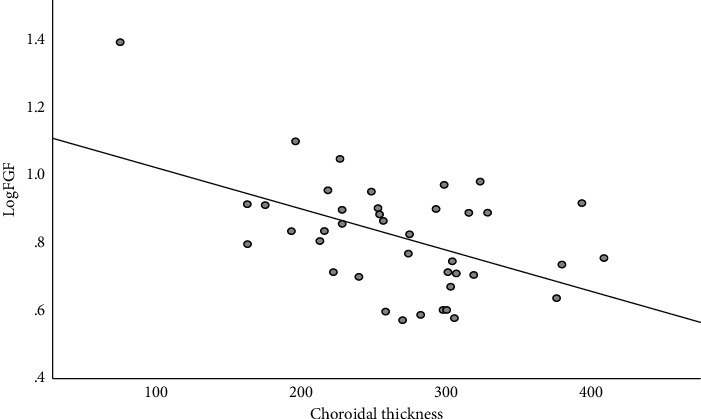
Correlation between aqueous humor FGF and CT in DME patients.

**Figure 4:**
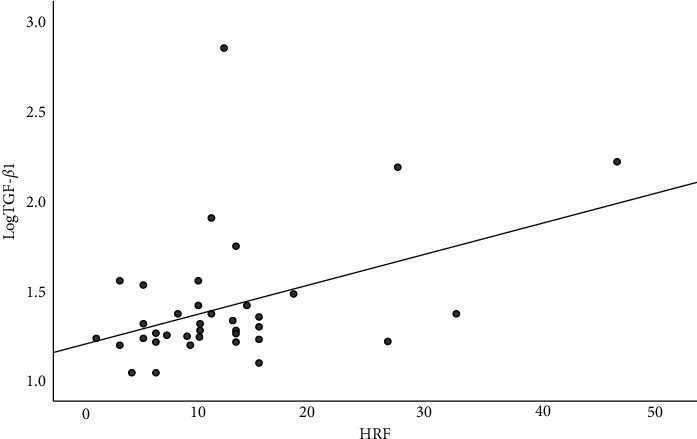
Correlation between aqueous humor TGF-*β*1 and HRF in DME patients.

Spearman correlation analysis revealed that the thinner the CT, the higher the aqueous humor IL-6 level (*r* = −0.313, *P* = 0.049, Figure 2) and the higher the aqueous humor FGF level (*r* = −0.361, *P* = 0.022, Figure 3); aqueous humor TGF-*β*1 was significantly correlated with HRF (*r* = 0.323, *P* = 0.045, Figure 4), and the higher number HRF suggested higher TGF-*β*1 levels. However, aqueous humor IL-6, IL-8, IL-10, VEGF, VCAM-1, ICAM-1, TGF-*β*1, FGF, and MCP-1 levels were not significantly correlated with CMT and MV. The number of HRF was significantly correlated with CMT (*r* = 0.451, *P* = 0.004) and MV (*r* = 0.541, *P* < 0.001), and not with CT (*r* = 0.248, *P* = 0.008).

The aqueous humor cytokine data were log-transformed to remove the impact of outliers and multiple linear regression analysis was carried out to better understand the relationship between aqueous humor cytokine levels and retinal choroidal structures. Univariate and multivariate linear regression analyses were performed for IL-6, FGF, and TGF-*β*1, separately (Table 3). The univariate regression analysis showed that CT (*P*=0.015), SRD (*P*=0.030), CME (*P*=0.032), and DRT (*P*=0.098) correlated with the increase of IL-6. The multivariate regression analysis showed that CT (*P*=0.002) and SRD (*P*=0.017) was significantly correlated with IL-6. The univariate regression analysis showed that CT (*P*=0.001), DM duration (*P*=0.091), total cholesterol (*P*=0.091), SRD (*P*=0.036), and CME (*P*=0.065) correlated with the increase of FGF. The multivariate regression analysis showed that CT(*P*=0.002) and SRD (*P*=0.005) was significantly correlated with FGF. The univariate regression analysis showed that CT (*P*=0.001), triglycerides (*P*=0.097), and HRF (*P*=0.008) correlated with the increase of TGF-*β*1. The multivariate regression analysis showed that triglycerides (*P*=0.030) and HRF (*P*=0.021) was significantly correlated with TGF-*β*1.

## 4. Discussion

Depending on the OCT morphology and pathogenesis, DME can manifest as SRD, DRT, CME, or mixed type. SRD mainly forms as the result of extraretinal barrier dysfunction; DRT presents as diffuse retinal edema mainly caused by retinal cell hypoxia and intracellular edema; CME primarily related to intraretinal barrier dysfunction and manifest as intraretinal fluid, muller cell, intracytoplasmic swelling, and necrosis [10, 11].

In this study, we found that IL-6 and FGF levels were significantly higher in DME patients with SRD than DME patients without SRD. A multivariate linear regression analysis showed that both aqueous humor IL-6 and FGF levels were associated with the presence of SRD. This result is consistent with previous studies, suggesting that inflammation plays an important role in the development of SRD. Abcouwer [12] reported that IL-6 was closely associated with SRD-type DME. Sonoda [13] et al. divided DME into groups with and without SRD, and demonstrated that IL-6 and IL-8 levels were significantly greater in eyes with SRD than in eyes without SRD, IL-6 levels were significantly associated with the presence of SRD after adjusting for age and sex. In one study, DME was divided into DRT, CME, and SRD types. The IL-6 levels between the three groups were also significantly different, with the SRD group having significantly higher IL-6 levels than the CME group [14]. IL-6 is a proinflammatory cytokine that regulates the immune response and initiates the acute phase response as a factor necessary for tissue injury healing. Injury-activated cytokines draw inflammatory cells to the site of injury, which stimulates the secretion of IL-6 and in turn induces the production of VEGF [15]. FGF is another growth factor that promotes cell proliferation, maintains tissue integrity, anticipated in wound healing, and is associated with the fibrotic process in DR [16]. Therefore, elevated levels of IL-6 and FGF in the SRD group may be associated with impairment of the outer retinal barrier, RPE cells and the choroid; however, the exact mechanism remains to be further investigated. Hwang et al. [17] reported that VEGF levels in aqueous humor were greater in the SRD group than in the no-SRD group. Our study was consistent with Hwang et al., as we demonstrated higher VEGF levels in DME patients with SRD than DME patients without SRD, although the difference was not statistically significant.

Previous studies found that IL-6 and IL-8 levels were significantly lower in CME eyes than in non-CME eyes [13]. However, the present study found that IL-6 levels in DME patients with CME were lower than those without CME, and IL-8 levels were higher in DME patients with CME were lower than those without CME, although the differences were not statistically significant. Meanwhile, this study showed that VEGF levels were significantly higher in DME patients with CME than in DME patients without CME. It has been reported that VEGF can induce conformational changes in the tight junctions of vascular endothelial cells and increase retinal vascular permeability, thus possibly causing intraretinal barrier dysfunction and extracellular edema between retinal layers [18]. Kim et al. [14] also reported significant differences in VEGF levels between the DRT, CME, and SRD groups; however, a post hoc comparison did not show statistically significant differences between any two groups.

Sonoda et al. [13] reported no significant differences in IL-6, IL-8, and VEGF levels between eyes with and without DRT in DME patients. The present study also found no significant difference in the VEGF levels between DME eyes with and without DRT. However, the IL-6, TGF-*β*1, and MCP-1 levels were significant greater in DME eyes with DRT than DME eyes without DRT, suggesting that inflammation plays an important role in macular diffuse edema.

HRF are thought to be activated microglia in the retina and are an imaging marker of retinal inflammation [19, 20]. Previous studies reported that HRF level was significantly greater in DME patients with SRD than in DME patients without SRD [20, 21]. TGF-*β*1 plays an important role in the process of angiogenesis, endothelial cell proliferation, extracellular matrix deposition, and tissue fibrosis [22]. We found that higher TGF-*β*1 levels were associated with greater HRF numbers. TGF-*β*1 has been reported to play a role in regulating microglia-mediated synaptic pruning in retina [23]. Therefore, TGF-*β*1 may promote migration and transformation of microglia, which were noted to manifest as HRF in DME.

Only a limited number of studies have analyzed the relationship between aqueous humor cytokines and CMT and MV. Hillier et al. [24] analyzed aqueous humor levels of VEGF, TGF-*β*, ICAM-1, IL-2, IL-3, IL-6, IL-8, IL-10, IL-17, VCAM-1, MCP-1, and EGF in 49 DME patients and analyzed their relationship with CMT and MV. A multiple regression analysis showed that only the increased level of ICAM-1 was found to be associated with an increase of MV, other cytokines were not significantly related to either the CMT or MV. Jonas et al. [25] measured macular thickness (defined as the distance between the inner retinal boundary membrane and the RPE) in 15 patients with DRT and found that central macular thickness was significantly associated with ICAM-1 levels. Kwon and Jee [26] analyzed the relationship between IL-1*β*, IL-2, IL-8, IL-10, IL-17, PlGF, and VEGF levels and CMT in 64 DME patients, and found that only IL-10 levels were correlated with CMT. In this study, we found that aqueous humor cytokine IL-6, IL-8, IL-10, VEGF, VCAM-1, ICAM-1, TGF-*β*1, FGF, and MCP1 levels lacked significant correlation with CMT or MV.

As far as we are aware, there are currently no research studies available that discuss the association between the CT and aqueous humor cytokine levels. The relationship between CT and the severity of diabetic retinopathy also remains unclear. Some studies reported that patients with diabetic retinopathy have higher CT than controls, while other studies reported that CT decreases as the severity of diabetic retinopathy increases or a lack of significant correlation between CT and diabetic retinopathy [27, 28]. Recently, it has been suggested that diabetic retinopathy causes an early increase in CT, which then decreases as diabetic retinopathy progresses. However, there was no statistically significant relationship between CT and DME. [29]. Gerendas et al. [30] studied 142 fluorescent angiographic images of patients with DME and showed that the thinner the CT, the greater the area of retinal leakage. In DME recurrence, Mathis et al. [31] discovered an increase in CT. In DME eyes with higher CT, Rayess et al. [32] discovered a better short-term morphological and functional response following anti-VEGF. All these studies suggest that the choroid may be involved in the pathogenesis of diabetic retinopathy and DME; however, their definite roles and mechanisms are not clear. In this study, we found that the thinner the CT, the higher the aqueous humor IL-6 and FGF levels. However, more studies are needed to confirm the exact association and to explore the mechanism.

There are several limitations in our current study. First, the number of patients included is relatively small, which may have attenuated the statistical power for detecting differences between the groups. Second, the vitreous fluid may be a better sample to reflect the posterior segment pathology than aqueous fluid. However, aqueous sampling is less invasive than vitreous fluid sampling and currently the most used intraocular specimen for the study of retinal diseases. Another limitation is a short follow-up, and the response to anti-VEGF treatment has varied in different studies and may be subjective in the present study.

In conclusion, this study suggested that significant macular cystoid edema changes may be related to high VEGF concentrations, and thin CT, the presence of SRD, or a high number of HRF on OCT macular scans in DME patients may indicate high levels of intraocular inflammatory factors. We believe that the reorganization of these characteristic OCT structures will facilitate medical professionals to estimate the levels of VEGF and intraocular inflammatory factors noninvasively in DME patients and to make better treatment strategies.

## Figures and Tables

**Table 1:** Comparison of baseline characteristics in patients with different types of DME.

	*SRD*		*P*	*CME*		*P*	*DRT*		*P*
	Without (*n* = 31)	With (*n* = 9)		Without (*n* = 8)	With (*n* = 32)		Without (*n* = 10)	With (*n* = 30)	
Age	63.3	60.0	0.363	61.7	62.7	0.804	61.1	63.0	0.584
Sex (F/M)	14/7	6/3	0.256	5/2	15/13	0.212	6/4	14/16	0.465
DM duration	14.2	14.7	0.847	14.5	15.1	0.832	14.9	13.8	0.671
HbA1c	7.6	7.9	0.715	7.7	8.3	0.448	7.8	8.5	0.548
Hb	129.3	128.1	0.829	127.7	130.9	0.548	130.7	121.3	0.044
BUN	7.8	6.6	0.228	7.0	6.4	0.619	7.2	5.5	0.121
Creatinine	101.1	75.2	0.082	81.3	81.2	0.994	84.8	65	0.241
Triglycerides	2.1	1.9	0.690	2.0	1.6	0.395	2.1	1.5	0.247
Total cholesterol	5.0	6.1	0.357	7.7	5.4	0.102	6.0	5.2	0.554
HDL cholesterol	1.2	1.3	0.551	1.3	1.2	0.548	1.2	1.3	0.843

DM, diabetes mellitus; Hb, hemoglobin; and BUN, blood urea nitrogen.

**Table 2:** Comparison of aqueous humor cytokine concentrations (pg/ml) and retinal choroidal structural parameters in patients with different types of DME.

	*SRD*		*P*	*CME*		*P*	*DRT*		*P*
	Without (*n* = 31)	With (*n* = 9)		Without (*n* = 8)	With (*n* = 32)		Without (*n* = 10)	With (*n* = 30)	
IL-6	**4.7 (7–20.8)**	**35.2 (8.3–45.1)**	**0.037**	12.9 (5.3–66.9)	8.6 (5–34.4)	0.521	**5.6 (3.7–11.4)**	**13.3 (6.1–35.6)**	**0.042**
IL-8	4.1 (6.2–17.7)	15.1 (9.8–36)	0.054	6.4 (3.8–29)	8.7 (4.6–18.9)	0.710	7.9 (3.4–19.3)	8.0 (4.6–20.8)	0.492
IL-10	2.6 (2.5–2.8)	2.9 (2.5–3.6)	0.331	2.8 (2.6–3.1)	2.6 (2.5–2.9)	0.437	2.7 (2.3–2.7)	2.7 (2.5–3.1)	0.200
VEGF	61.4 (26.9–101.2)	110.8 (61.5–135.2)	0.078	**33.4 (11.8–51.3)**	**84 (52.5–130.2)**	**0.005**	62.5 (22.6–97.9)	71.3 (38.2–123.5)	0.399
VCAM-1	1720.5 (737.3–2436.2)	2258 (1278.3–3309.7)	0.169	2340.5 (899.6–4023.1)	1750.6 (746.3–2453.4)	0.295	972.0 (407.8–1974.7)	1829.2 (995.5–2980.3)	0.070
ICAM-1	728.6 (455.6–1079.4)	651.1 (523.7–1222.4)	0.859	749.1 (383.4–1337.7)	699.8 (486.8–1058.8)	0.761	611.1 (139.5–1413.1)	730.4 (566.4–1017.5)	0.333
TGF-*β*1	19.2 (17–23.6)	26.4 (17.7–46)	0.145	21.9 (17.5–68.1)	19.6 (16.9–24.0)	0.327	**17.6 (16.4–21.0)**	**22.1 (17.3–34.8)**	**0.039**
FGF	**6.3 (5.1–7.9)**	**8.3 (6.2–9.5)**	**0.048**	5.8 (4.5–11)	7.0 (5.2–8.1)	0.565	6.9 (5.5–7.9)	6.4 (4.6–8.2)	0.755
MCP-1	610.9 (434.2–954.4)	717.1 (474.7–939.4)	0.611	767.2 (339.5–863.3)	614.2 (459.2–969.4)	0.684	**380.5**	**642.3**	**0.014**
CMT (*μ*m)	375 (324–517)	495 (372–571)	0.116	331 (292–460)	435 (340–545)	0.073	361 (329–497)	426 (333–530)	0.851
MV (mm^3^)	0.29 (0.26–0.41)	0.39 (0.29–0.45)	0.116	0.27 (0.23–0.36)	0.35 (0.27–0.43)	0.081	0.28 (0.26–0.39)	0.34 (0.26–0.42)	0.827
CT (*μ*m)	260.4 ± 60.9	277.6 ± 87.4	0.506	**221.5** **±** **89.4**	**275.0** **±** **56.9**	**0.042**	258.3 ± 44.5	266.3 ± 73.4	0.749
HRF	**9.5 (5.8–13)**	**15 (13.5–29)**	<**0.000**	9.5 (4.5–12.5)	11 (6–15)	0.402	**6 (5–9.5)**	**13 (9.5–15)**	**0.004**

Bold values: significant p-values (*p* < 0.05).

**Table 3:** Univariate and multivariate linear regression analysis for IL-6, FGF, and TGF-*β*1.

	*Log (IL-6)*				*Log (FGF)*				*Log (TGF-β1)*			
	*Univariate analysis*		*Multivariate analysis*		*Univariate analysis*		*Multivariate analysis*		*Univariate analysis*		*Multivariate analysis*	
	Coefficient	*P*value	Coefficient	*P*value	Coefficient	*P*value	Coefficient	*P*value	Coefficient	*P*value	Coefficient	*P*value
Age	0.008	0.997			0.004	0.121			0.004	0.542		
CT	−0.002	0.015	−0.003	**0.006**	−0.001	0.001	−0.002	**0.002**	−0.001	0.100	−0.002	0.068
DM duration	0.008	0.426			0.006	0.091	−0.003	0.400	0.002	0.797		
Hb1c	0.060	0.257			0.019	0.343			0.013	0.759		
BUN	0.040	0.217			0.005	0.680			−0.007	0.799		
Creatinine	0.003	0.159			0.001	0.225			0.002	0.311		
Triglycerides	0.020	0.781			0.018	0.493			−0.090	0.097	−0.120	**0.030**
Total cholesterol	0.030	0.245			0.018	0.058	0.017	0.053	0.007	0.728		
HDL cholesterol	0.197	0.364			0.072	0.376			0.059	0.732		
CMT	0.000	0.659			0.000	0.871			0.000	0.758		
MV	0.286	0.632			0.03	0.892			0.169	0.720		
SRD	0.356	0.030	0.347	**0.017**	0.128	0.036	0.170	**0.005**	0.108	0.418		
CME	−0.388	0.032	−0.267	0.089	−0.125	0.065	−0.020	0.762	−0.131	0.368		
DRT	0.265	0.098	0.206	0.133	0.030	0.620			0.158	0.214		
HRF	0.010	0.250			0.004	0.182			0.017	0.008	0.015	**0.021**

Significant values with *P* < 0.05 in multivariate analysis are in bold.

## Data Availability

The data used to support the findings of this study are available from the corresponding author upon request.
